# A novel glycosylated anti-CD20 monoclonal antibody from transgenic cattle

**DOI:** 10.1038/s41598-018-31417-2

**Published:** 2018-09-04

**Authors:** Ran Zhang, Chenjun Tang, Huaizu Guo, Bo Tang, Sheng Hou, Lei Zhao, Jianwu Wang, Fangrong Ding, Jianmin Zhao, Haiping Wang, Zhongzhou Chen, Yunping Dai, Ning Li

**Affiliations:** 1 State Key Laboratory for Agrobiotechnology, College of Biological Sciences, China Agricultural University, Beijing, 100194 China; 2 State Key Laboratory of Antibody Medicine and Targeted Therapy, Shanghai Key Laboratory of Cell Engineering, Shanghai, 200433 China; 3 Wuxi KGBIO biotechnology Limited Liability Company, Wuxi, 214145 China; 4 National Clinical Research Center for Normal Aging and Geriatric, Institute of Geriatric, PLA General Hospital, Beijing, 100853 China

## Abstract

The monoclonal antibody (mAb) against CD20 known as Rituxan is widely used to treat autoimmune diseases and lymphomas. However, further application of Rituxan faces challenges of high production cost, which limits its availability in developing countries. Here, we report a new approach for large production of a recombinant anti-CD20 mAb in the milk of transgenic cattle (at a yield of up to ~6.8 mg/mL), with ~80% recovery rate and >99% purity. Crystallography study showed that our recombinant mAb is structurally nearly identical to Rituxan with only minor differences in N-linked glycosylation pattern. Functional study showed that, while our mAb shared similar target-cell binding capacities and complement-dependent cytotoxicity with Rituxan, our product exhibited a higher binding affinity for FcγRIIIα and a greater antibody-dependent cellular cytotoxicity. Accordingly, our recombinant mAb demonstrated a superior efficacy over Rituxan against B-cell lymphomas in severe combined immunodeficiency mice. Taken together, our data supports transgenic cattle as a novel model for cost-competitive, large-scale production of therapeutic antibodies.

## Introduction

In 1997, Rituxan was approved by the US Food and Drug Administration (FDA) as the first therapeutic recombinant monoclonal antibodies (mAb) to treat non-Hodgkin lymphoma^1^. Rituxan is a chimeric immunoglobulin G1 (IgG1) mAb against the human CD20 antigen, which is uniquely expressed on B cell surface. Previous studies found that Rituxan acts through several mechanisms contributing to tumor clearance. This includes complement-dependent cytotoxicity (CDC), antibody-dependent cell-mediated cytotoxicity (ADCC), and the direct induction of programmed cell death, which together can effectively reduce the circulating B-cell counts in patients with lymphoma^2,3^.

The clinical success of Rituxan has significantly increased the market demand and generated huge profits, making Rituxan one of the top 10 best-selling antibody drugs with over $7.5 billion annual sale worldwide in 2014. Rituxan therapies require generally high doses, leading to high demand in Rituxan manufacturing capacity^4^. Rituxan is currently produced via mammalian cell culture, which is high cost and thus limits its mass production capacity^5^. On the other hand, patient population that need anti-CD20 mAb treatment remains large. For example, in China the incidence of non-Hodgkin lymphoma has increased sharply in recent years. However, the penetration rate of Rituxan is less than 10% in China owning to its high cost. Therefore, the development of new methods for enhanced mAb production capacity is highly demanded.

Over the past decade, recombinant anti-CD20 mAbs have been successfully produced in silkworms and plant seeds, but neither of these hosts has enabled large-scale production that meets the market demand^6–8^. Mammary gland bioreactors derived from transgenic animals have been considered as efficient and attractive systems for producing recombinant pharmaceutical proteins, because their extremely high production capacities with low manufacturing costs^9^. Eukaryotic proteins, particularly those from mammalians, undergo complex post-translational modifications, such as glycosylation, which can widely vary among cell types and play a major role in protein functions^10,11^. Noteworthy, a recombinant human anti-thrombin III antibody (ATryn) that is produced in milk of transgenic goats and a C1 esterase inhibitor (Ruconest) that is produced in milk of transgenic rabbits have been approved for commercial use by the European Medicines Agency and the US FDA. These approved products demonstrate the commercial ability and broad market potential of mAbs mass-produced by transgenic animals^12–14^.

Here, we reported a new approach to cost-effectively produce recombinant anti-CD20 mAb in large scale using the mammary gland bioreactors of transgenic cattle. The mAb can be easily recovered from milk to a high purity. The recombinant mAb shared similar structure, antigen-binding capacity and CDC effect with Rituxan, but different in glycosylation pattern. Importantly, this different glycosylation may contribute to the observed higher ADCC effects and *in vivo* therapeutic efficacy.

## Results

### Generation of transgenic cattle expressing an anti-CD20 mAb

**Figure 1:**
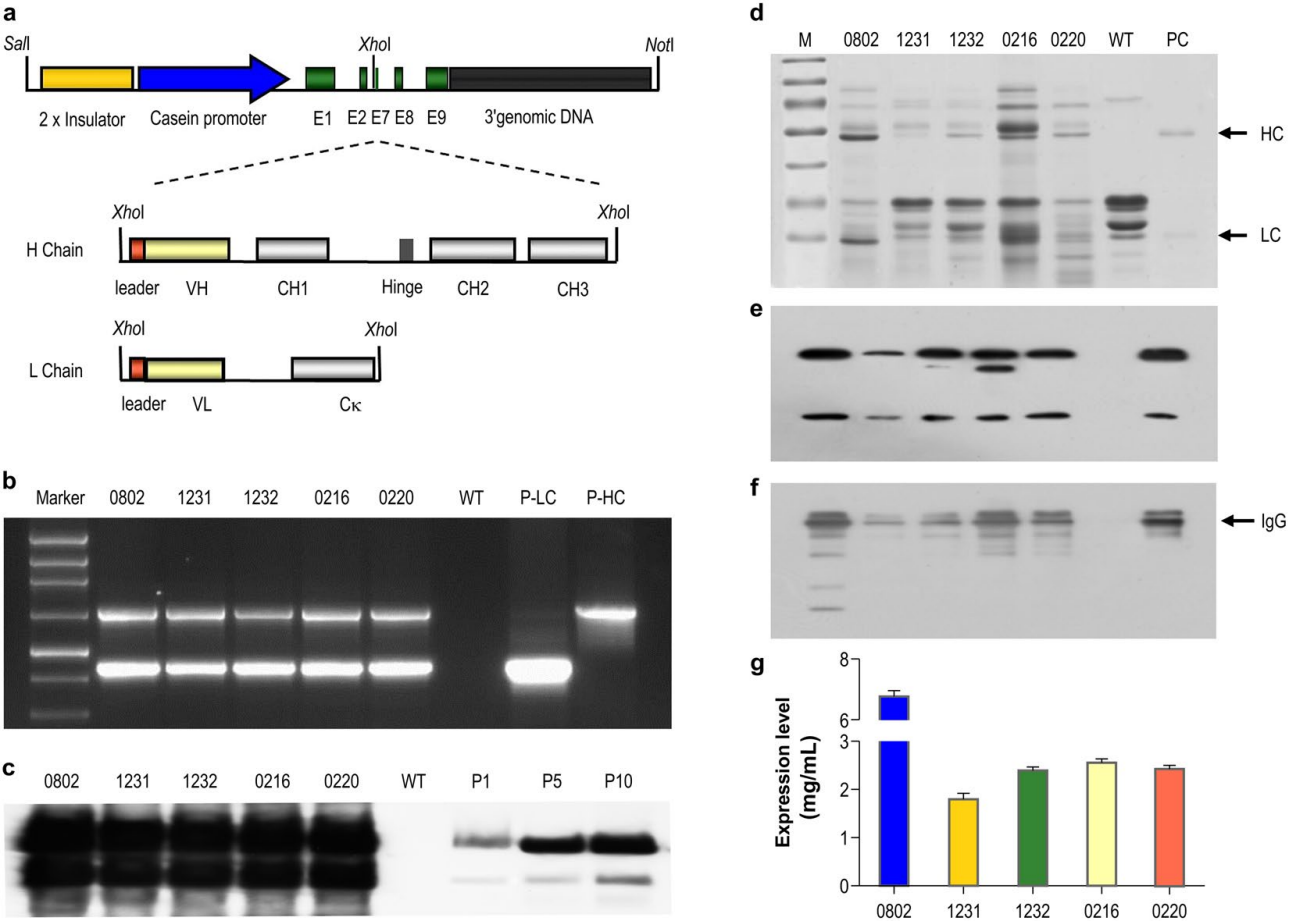
Generation of transgenic cattle expressing a recombinant anti-CD20 mAb in their milk. (**a**) Schematic illustration of the two transgene constructs encoding heavy chain (17.2 kb) and light chain (16.5 kb) of the recombinant anti-CD20 mAb, respectively. The transgene construct backbone also contains sequences coding for the following elements: 2× chicken β-globin insulator (2.4 kb); the goat β-casein promoter (4.1 kb); untranslated exon (E)1; parts of E2, E7, E8, and E9 (3.7 kb); and the 3′ genomic DNA of β-casein (5.5 kb). The dotted lines indicate the insertions of the HC- and LC-encoding sequences into the unique *XhoI* restriction site. The hatched boxes represent the IgG secretory leader sequence, the variable regions (VH and VL), and the constant regions indicated by CH1, Hinge, CH2 and CH3 for HC, or Cκ for LC. The relevant restriction enzymes sites are indicated. (**b**) PCR detection of the transgenes in transgenic cattle. Marker, 1-kb DNA ladder; P-LC, positive plasmid containing the LC-encoding sequences; P-HC, positive plasmid containing the HC-encoding sequences; WT, genomic DNA of wild-type cattle; genomic DNA of five transgenic cattle lines were indicated by 0802, 1231, 1232, 0216, and 0220. The amplified products for the HC- or LC-encoding sequences were 1.5 kb and 0.8 kb, respectively. (**c**) Southern blot analysis of transgenic cattle. The digested genomic DNA was hybridized with the probe mixture of the HC- and LC- encoding fragments. The hybridization signals for HC and LC are indicated. P1/P5/P10, signal intensities of the positive plasmid DNA controls equivalent to 1, 5, and 10 gene copies, respectively. (**d**–**f**) Expression of the recombinant anti-CD20 mAb in the milk of transgenic cattle, as characterized by SDS-PAGE (**d**) and western blotting under reducing (**e**) and non-reducing conditions (**f**). WT, milk from wild-type cattle. PC, purified human IgG. (**g**) Recombinant anti-CD20 mAb expression levels in milk from different transgenic cattle were detected by enzyme-linked immunosorbent assays.

Two cassettes, each containing the IgG heavy chain and light chain genes under the control of the goat β-casein promoter were constructed to generate transgenic cattle that expressed the anti-CD20 mAb in milk (Fig. 1a). Two positive cell lines (FOV-3 and BFF-6) were used to produce cloned embryos for somatic cell nuclear transfer (SCNT). Ten calves were born while 5 calves, referred as #0802, #1231, #1232, #0216, and #0220, remained healthy during the study. Among them, one (#0802) developed with the FOV cell type, whereas the remaining four developed with the BFF cell type (Supplementary Fig. 1a). PCR and Southern blotting detected both the HC and LC transgenes in the genome of all five calves (Fig. 1b,c). Next-generation sequencing showed a unique transgene-integration site in the intergenic region of chromosome 3 with the copy numbers of 20 for both the HC and LC transgenes (Supplementary Fig. 1b). A fluorescence *in situ* hybridization (FISH) analysis detected a single integration site of the transgenes, confirming the sequencing results (Supplementary Fig. 1c,d).

In order to obtain the qualitative results on whether the recombinant mAb was expressed and whether they assembled and modified correctly, the milk samples were analysed by SDS-PAGE and western blotting (Fig. 1d–f). Two specific bands representing the HC (50 kDa) and LC (25 kDa), respectively, were detected under reducing conditions. In addition, strong 150-kDa bands under non-reducing conditions were also detected, indicating that the recombinant mAb was assembled correctly in the transgenic cattle. The mAb expression levels were all higher than 2 mg/mL, with the highest one being 6.8 mg/mL (Fig. 1g). Furthermore, the phenotype and the expression pattern of two offspring (#1321 and #1325) from transgenic cattle #0220 were also detected, demonstrating that the transgene was stable between generations (Supplementary Fig. 2a–c). Transgenic cow #0220 was used for the subsequent studies.

### Purification of the anti-CD20 mAb from the transgenic milk

**Figure 2:**
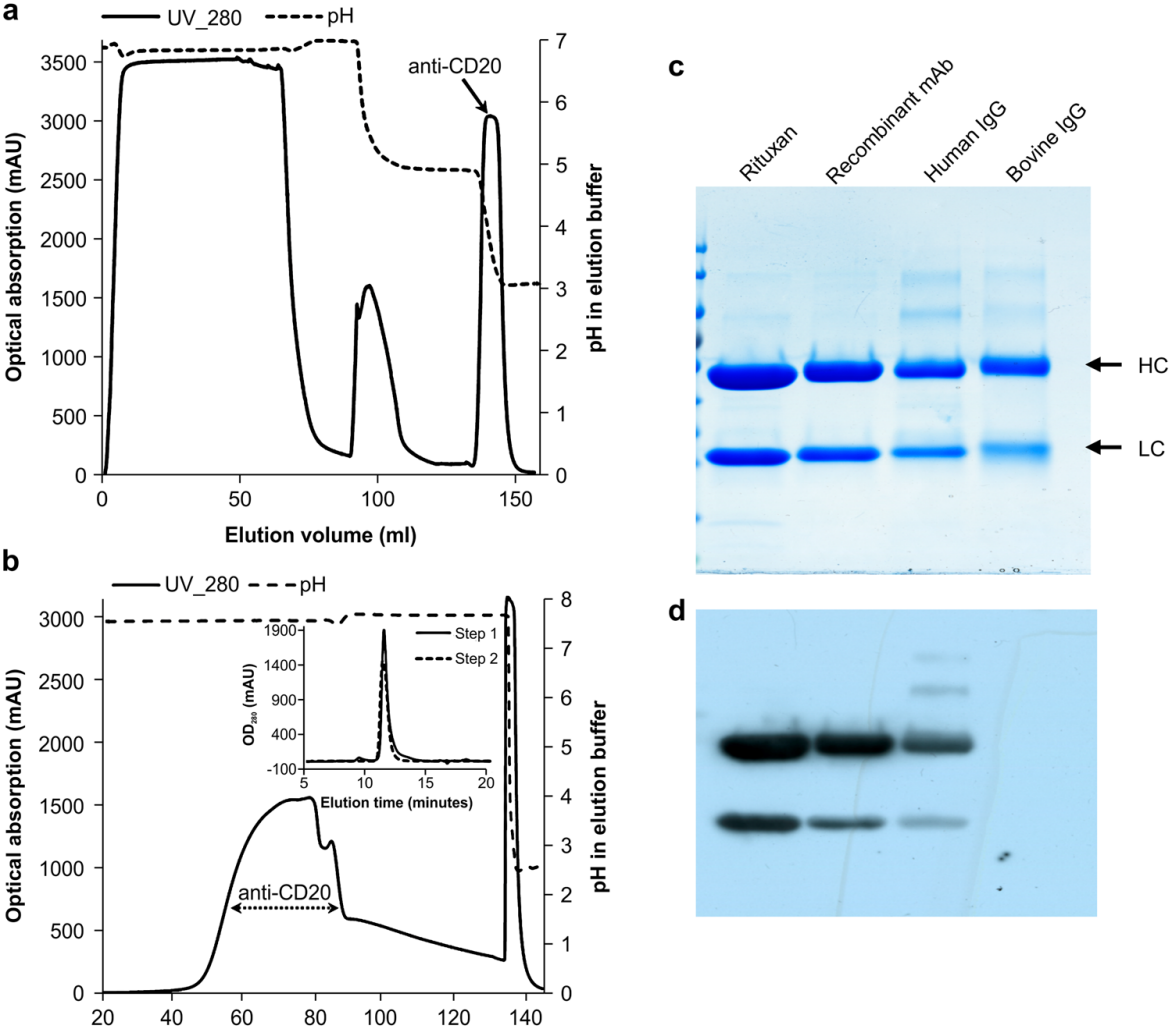
Purification of the recombinant anti-CD20 mAb. (**a**) The initial step of mAb using Protein A affinity chromatography. (**b**) The second step of mAb capture used anion-exchange chromatography, and purity was analysed by SEC-HPLC. (**c**,**d**) SDS-PAGE (**c**) and western blotting (**d**) analysis of the purified anti-CD20 mAb.

A scaled-up purification procedure was developed to purify mAb for non-clinical applications, using two chromatography steps with MabSelect and Capto media, followed by concentration/desalting and sterile filtration. The result showed that the anti-CD20 mAb was eluted at pH 3.5 from a MabSelect SuRe LX column, differing from the bovine antibodies that were eluted at pH 5.0 (Fig. 2a). The SEC-HPLC revealed a defined anti-CD20 mAb peak with a purity of 93.6 ± 7% and a recovery rate of 95% ± 4%. The mAbs were further purified by anion-exchange chromatography, reaching a final purity of 99.8 ± 0.1% with the recovery rate of 78.6 ± 0.1% (Fig. 2b). The SDS-PAGE and western blotting showed define bands corresponding to the HC and LC proteins, indicating that our product was highly stable after this purification procedure and that the purity of our mAb was resemble Rituxan (Fig. 2c,d). This highly purified anti-CD20 mAb was used in the subsequent functional characterization.

### Sequences and glycosylation of the recombinant anti-CD20 mAb

**Figure 3:**
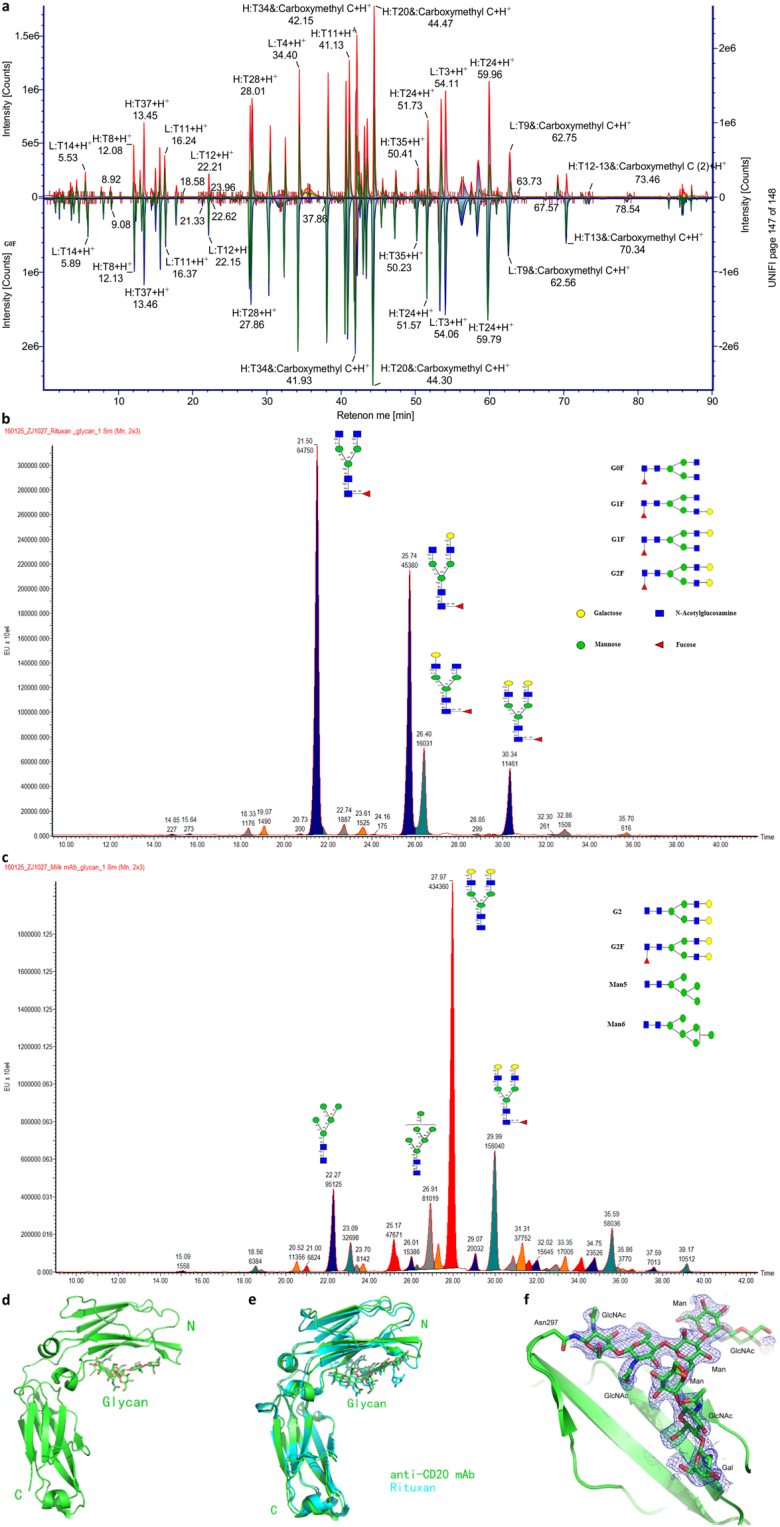
Characterization of the N-glycosylation patterns and crystal structures of the recombinant anti-CD20 mAb and Rituxan. (**a**) Comparison of the LC-MS peptide-mapping results of the recombinant mAb and Rituxan. The upper panel with red peaks represents the peptide peak assignment of the recombinant mAb, while the bottom panel with green peaks represents those of Rituxan. (**b**,**c**) Comparison of the oligosaccharide profiles of Rituxan (**b**) and the recombinant mAb (**c**), as analysed by MS. The major peaks in the spectra are labelled with the oligosaccharide structure assignments. (**d**) Overall structure of the recombinant mAb Fc fragment. The glycosylated glycan is shown in stick representation. (**e**) Structural superimposition between the recombinant mAb Fc fragment (green) and Rituxan Fc fragment (PDB: 1L6X). (**f**) The “omit” electron density map of the glycosylation site at Asn 297 in the recombinant mAb Fc fragment, contoured at 1.0 σ.

Peptide mapping analyses of the recombinant mAb produced in milk and Rituxan by LC-MS showed that our mAb shared nearly identical amino acid sequences with Rituxan (Fig. 3a). However, 2 HC allotypes were found, in one the amino acid residues of KAE (218–220) in Rituxan were replaced with RVE in our mAb, while in the other the residues of DEL in Rituxan (360–362) were replaced by EEM (Supplementary Fig. 3).

The N-glycan profiles of our mAb and Rituxan were also determined by LC-MS (Tables S1 and S2). N-glycosylation was only observed at the conserved Asn297 site for both mAbs. For Rituxan, peak integration revealed that G0F (core-fucosylated biantennary N-glycans without galactose), G1F (with 1 galactose residue) and G2F (with 2 galactose residues) contributed 44.08%, 41.33%, and 7.71% to the total glycans, respectively, which matched the typical main N-glycan profiles of IgG derived from CHO cells (Fig. 3b). It was noted that the afucosylated glycans only accounted for 2.27 ± 0.06% of Rituxan glycans. In contrast afucosylated glycans were the major N-glycans in our recombinant mAb, representing up of 83.19 ± 0.23% of the total glycans. The core fucose residue was removed from the oligosaccharides attached to Asn297, and the G2 glycans were dominant, accounting for 36.04% of the total glycans (Fig. 3c). In addition, the mannose-type glycans in our recombinant mAb were more prevalent (~27%) than those observed in Rituxan.

### Structure of the recombinant anti-CD20 mAb

The purified Fc fragment of our mAb was further studied by crystallography to characterize its structural features. Two different space groups were obtained: *C*222_1_ and *P*2_1_2_1_2_1_. Both structures were solved using molecular replacement and refined to a resolution of 2.05 Å and 2.34 Å, respectively (Table S3). Both structures of the anti-CD20 Fc fragment were in good agreement with the previously solved structure of the human IgG1 Fc fragment (PDB: 1L6X)^15^, with a root-mean-square deviation (RMSD) of 1.0 or 1.1 Å between their C-alpha atoms (Fig. 3d,e). Importantly, the glycosylation pattern of our anti-CD20 mAb was identified, which comprises mainly the G2 glycan type, consistent with the LC-MS results. Moreover, no fucose was observed in the electron density map (Fig. 3f). Interestingly, only one of the galactose residues at the end of the G2 glycan was observed due to its interaction with the anti-CD20 mAb.

### Functional activity of the recombinant anti-CD20 mAb

**Figure 4:**
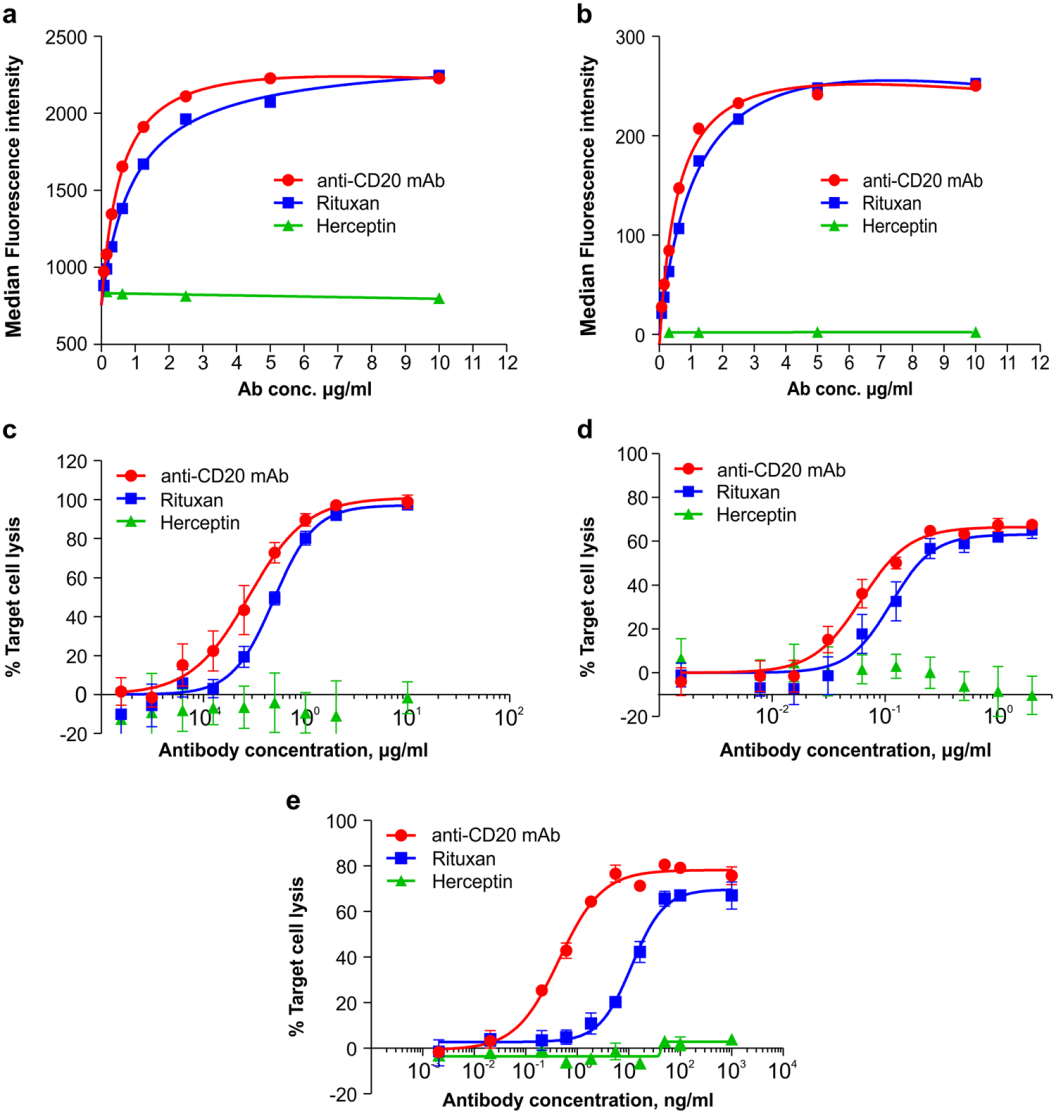
Functional evaluation of the recombinant anti-CD20 mAb *in vitro*. (**a**,**b**) CD20-binding activity of the recombinant mAb. Daudi (**a**) and Raji (**b**) cells were incubated with serial log dilutions of the mAbs, and their binding activities were assayed by flow cytometry using a FITC-conjugated goat anti-human IgG (H + L). Data are presented as the mean ± SD (n = 3). (**c**,**d**) CDC induced by the anti-CD20 mAb. Daudi (**c**) and Raji (**d**) cells were incubated with increasing concentrations of mAbs in the presence of human serum. CDC activity was measured using a Luminescent Cell Viability Assay Kit. Data are presented as the mean ± SD (n = 3). (**e**) ADCC induced by the anti-CD20 mAb. ADCC activity against Raji cells was determined using human PBMCs as effector cells at an effector-to-target cell ratio of 5:1. The activity was measured using a standard LDH assay. Data are presented as the mean ± SD (n = 3).

The biological activities of our anti-CD20 mAb were analysed through independent approaches using 2 CD20^+^ human lymphoma cell lines (Daudi and Raji). Specific antigen-binding assay revealed that our recombinant mAb bound to Daudi and Raji cells in a dose-dependent manner, with an antigen-binding activity similar to that of Rituxan (Fig. 4a,b). In addition, the CDC mediated by our recombinant mAb against both Daudi and Raji cells was comparable to that of Rituxan (Fig. 4c,d).

As previous data showed that the absence of an N-glycan-containing fucose was related to a higher affinity for FcγRIIIa and ADCC activity, the binding affinity of the anti-CD20 mAb for FcγRs was measured. The *K*_D_ values between our mAb and FcγRIIIa-Val158 or FcγRIIIa-Phe158 were both dramatically reduced compared with those of Rituxan, indicating that the binding affinities of the recombinant anti-CD20 mAb for FcγRIIIa were much higher than those of Rituxan (Supplementary Fig. 4a–d and Table S4). In addition, we investigated whether natural killer (NK) cells, the key effector cells for ADCC, could enhance the ADCC activity of the recombinant mAb. The results showed that our recombinant mAb and Rituxan were both capable of eliciting strong ADCC activity against Raji cells in a dose-dependent manner (Fig. 4e). The ADCC activity of our mAb was significantly higher than that of Rituxan, as the EC_50_ for target cell lysis by Rituxan was 11.48 ng/mL, which was 26-fold greater than that of our mAb. These data suggested that the absence of fucose in the G2-type glycosylation pattern was critical for the enhanced activity of the recombinant anti-CD20 mAb.

### Therapeutic efficacy of the recombinant anti-CD20 mAb

**Figure 5:**
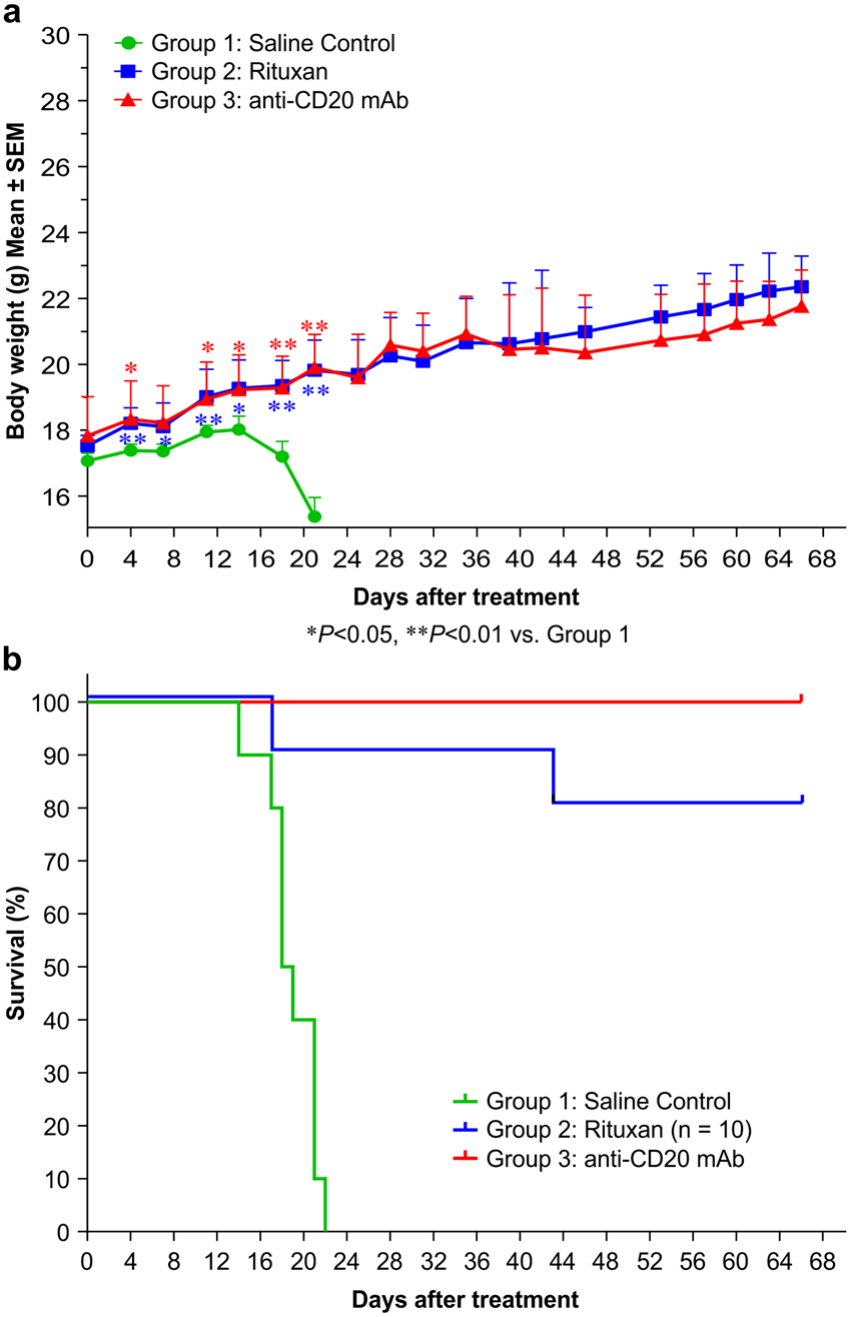
Therapeutic efficacy of the recombinant anti-CD20 mAb in treating B-cell lymphomas. Groups of 10 SCID mice were intravenously injected with 4 × 10^6^ Raji cells. Seven days after tumour cell inoculation, the mice were treated with 100 μg/mouse of the recombinant mAb, Rituxan, or a saline control. The mice were monitored daily and euthanized at the onset of hind leg paralysis. (**a**) The body weight curve of tumour-bearing SCID mice treated with the anti-CD20 mAbs. *p < 0.05, **p < 0.01. (**b**) Survival curve of the tumour-bearing SCID mice treated with the anti-CD20 mAbs.

Severe combined immunodeficiency (SCID) mice bearing Raji tumours (SCID/Raji) were used as B-cell lymphoma model to evaluate the therapeutic efficacy of the recombinant anti-CD20 mAb. After treated with our recombinant mAb (100 μg/mouse), Rituxan (positive control) or saline (negative control), mice in the saline group decreased their body weights dramatically starting at 14 days after administration. By contrast, mice in our mAb and Rituxan groups increased their body weights over time (Fig. 5a). Furthermore, the median survival time of mice in the saline group was only 18.5 days, whereas mice in the Rituxan and our recombinant mAb groups showed significantly improved survival times (p ≤ 0.0001 for each, compared with the saline control). It was noted that two mice died in the Rituxan group, one on day 17 and another on day 43 after tumor inoculation, whereas all mice in our mAb group remained healthy during the 66 day-experimental period (Fig. 5b). These results indicated that both Rituxan and our recombinant mAb exhibited potent anti-tumour activity, protecting the SCID mice against Raji tumours. Our data also indicated that our recombinant anti-CD20 mAb is more potent than Rituxan in providing this protection, probably due to their difference in glycosylation.

## Methods

### Cell Lines, antibodies, and animals

Two human Burkitt’s lymphoma cell lines, Daudi and Raji, were obtained from the American Type Culture Collection (Manassas, VA). Rituxan and Herceptin (Roche, Shanghai, China) were used as positive and negative controls, respectively, in both *in vitro* and *in vivo* assays. SCID-beige mice were housed under specific pathogen-free conditions. All methods were performed in accordance with relevant guidelines and regulations. All experiments were approved by the Institutional Animal Care and Use Committee of China Agricultural University (Permit Number: SKLAB-2012-06-01).

### Generation of transgenic cattle

Both the HC and LC DNA sequences of the anti-human CD20 antibody were synthesized and then two mammary gland-specific plasmids, pBC-HuG1F and pBC-HuK1F, were constructed as previously described^16^. The pBC1 plasmid was a commercialized expression vector which used the goat β-casein promoter to drive recombinant proteins expression specifically in the mammary gland duration lactation. The HC and LC transgenes were released from the plasmids by *Not*I and *Sal*I digestion, leading to a 17.2 kb and a 16.5 kb DNA fragments, respectively. After purification, the two fragments were used for transfection and the transgenic cloned calves were produced using the SCNT procedure, as previously described^17^.

### Transgene detection

Genomic DNA was isolated from an ear punch of each newborn calf, according to standard protocols. The transgene was detected by PCR using the primers pBC-F (5′-GATTGACAAGTAATACGCTGTTTCCTC-3′) and pBC-R (5′-CATCAGAAG TTAAACAGCACAGTTAG-3′) that were specific to β-casein intron 1 and 7 sequences and could therefore detect both the HC and LC genes simultaneously. After *Bgl*II digestion, genomic DNA (10 μg) was analysed by Southern blotting using hybridization probes that contain the HC and LC genes, respectively. The transgene integration sites and copy numbers were analysed by whole-genome sequencing, as previously described^18^.

### Milk sample collection

Milk samples were collected on indicated days throughout the first 3 months of early lactation from the transgenic and wild-type cattle. Skim milk was made via ultracentrifugation at 150,000 × *g* for 1 h at 4 °C to remove the casein micelles. Supernatant (whey fraction) was stored at −80 °C.

### Immunodetection of anti-CD20 mAb

Whey was separated on 12% SDS-PAGE gels under both reducing and non-reducing conditions. Anti-CD20 mAb was detected using an HRP-conjugated goat anti-human Fab-specific antibody (Sigma, St. Louis, MO) and ECL Western blot reagents (GE Healthcare). Purified human IgG (Sigma) was used as a positive control.

### Anti-CD20 mAb purification

After adjusting the pH to 4.6 with phosphoric acid, milk containing anti-CD20 mAb was centrifuged at 2,800 × *g* for 20 min to remove the caseins. Following a filtration through a 0.45-μm-thick ceramic membrane the whey was applied to a MabSelect SuRe LX affinity column and then a Capto-adhere column (GE Healthcare). The purified mAb was concentrated and examined for purity by SDS-PAGE, western blotting, and SEC-HPLC (Agilent Technologies, Santa Clara, CA). The purified anti-CD20 mAb was digested by papain (1:25, w:w) to obtain the Fc fragment for crystallography. The contaminated papain was removed via a His-affinity column, followed by an S-Sepharose ion-exchange column (GE Healthcare). After dialysis, the flowed through Fc fragment was further purified by SEC using a Superdex 75 column (GE Healthcare). Fractions containing the target mAb were pooled and concentrated to 8 mg/mL.

### Peptide-mapping and oligosaccharide profile analysis

Peptide-mapping analysis and N-linked glycans profiling of the anti-CD20 mAbs was performed as previously described^19^. The collected LC-MS/MS data for peptide-mapping were processed using BiopharmaLynx 1.3 software. The labelled glycans were separated on a HILIC column and detected using an ACQUITY UPLC fluorescence detector plus quadrupole time-of-flight (Q-TOF) MS.

### Crystallization and data collection

The anti-CD20 mAb Fc fragment was crystallized at 18 °C using the sitting-drop vapour-diffusion method. Crystals of the free-form Fc grew in a drop containing 1 μL of the protein solution and 1 μL of the reservoir solution (30% PEG 400, 0.1 M NaAc, pH 4.6, and 0.1 M CdSO_4_) over 4 days. The crystals were transferred to the cryoprotectant of the reservoir solution supplemented with 20% glycerol and then flash-cooled in liquid nitrogen before diffraction data collection. The diffraction data were collected at the BL17U1 or BL18U1 beam line at the Shanghai Synchrotron Radiation Facility. The data were indexed, integrated, and scaled using HKL2000 software^20^.

### Structure determination and refinement

An initial molecular-replacement solution was obtained using BALBES^21^ software and the structure of the human IgG1 Fc fragment (PDB 1L6X)^15^ as a model. The model was manually constructed using Coot software^22^, and refinement was performed with REFMAC5^23^. Two different space groups were obtained: *C222*_1_ and *P*2_1_2_1_2_1_. The figures in this article displaying molecular structures were generated using PyMOL software.

### Binding assay

Daudi and Raji cells were incubated with serially diluted anti-CD20 mAb at 37 °C for 1 h. The cells were incubated with a FITC-conjugated goat anti-human IgG (H + L) (Abcam, Cambridge, MA) for 1 h at 4 °C and analysed with a FACSCalibur system (BD Biosciences, San Jose, CA). The median fluorescence intensity was calculated using WinMidi2.9 software, and the Kd was calculated using GraphPad Prism software. Rituxan was used as a positive, while Herceptin was a negative control.

### Surface plasmon resonance (SPR) assay

To determine the affinity of Rituxan and our mAb for human FcγRIIIa, two soluble ectodomains of human CD16a (Phe158 and Val158) were immobilized on Series S CM5 chips by amine coupling, resulting in a density of 560 RU and 260 RU, respectively, with a Biacore T200 SPR system (Biacore, GE Healthcare). Seven different concentrations of Rituxan (ranging from 21,870–30 nM; serial threefold dilutions) and six different concentrations of our mAb (ranging from 810–3.333 nM; serial threefold dilutions) were injected through the flow cells, respectively. Background binding to the blank immobilized flow cells and buffer-only injections were subtracted, and the affinity constants were calculated using Biacore T200 Evaluation software and the 1:1 Langmuir binding model.

### CDC assay

Daudi and Raji cells were incubated with serially diluted anti-CD20 mAbs for 30 min. Following the incubation, normal human serum (10% vol/vol) was added as a source of complement. The cells were assayed using a CellTiter-Glo Luminescent Cell Viability Assay Kit (Promega, Madison, WI), and the data were read using PHERAStar Plus (BMG Labtech). Individual EC_50_ values were calculated using GraphPad Prism software.

### ADCC assay

The ADCC activity of the anti-CD20 mAb was measured by LDH-releasing assays using an LDH Kit (Roche) according to the manufacturer’s instructions. Briefly, Raji cells were incubated with serially diluted anti-CD20 mAb, followed by the addition of the NK/92/CD16a (158 v/v) effector cells at an effector-to-target ratio of 5:1. The LDH activity in the cell culture supernatants was measured using a FlexStation 3 microplate reader (Molecular Devices, Sunnyvale, CA). The maximum LDH release was determined by lysing the cells in 1% Triton X-100. Individual EC_50_ values were calculated using GraphPad Prism software.

### Immunotherapy

45 female SCID mice at 6- to 8-week of age were injected with 4 × 10^6^ Raji cells via the tail vein. 30 mice with confirmed successful injection were randomized assigned to 3 groups. After 7 days, the mice were treated with a single dose of antibody at 100 μg/mouse or saline as controls by intravenous injection. The mice were monitored and weighted daily and euthanized at the onset of hind leg paralysis. Data were analysed using Student’s *t*-test. The statistical significance level was set at *P* < 0.05. The survival curve was evaluated using the log-rank (Mantel-Cox) test.

## Discussion

As the first FDA-approved mAb, Rituxan has offered an effective treatment against most B-cell lymphoproliferative disorders over the past 30 years. However, known limitations of Rituxan, especially its high manufacturing cost, prevented its wide implementation, particularly in developing countries. Several previous studies have been conducted, trying to address these issues, including producing recombinant anti-CD20 mAbs in silkworms and plant seeds, but none has substantially reduced the manufacturing cost and patient population that need anti-CD20 mAb treatment remains large. Transgenic animal species, such as rabbits, goats and cows, offer attractive strategy to produce pharmaceutical proteins in their milk and cows are potentially the most appropriate species for their higher milk production capacity compared to others^24^. In this study, high level of recombinant anti-CD20 mAb production was seen in milk of the transgenic cattle, reaching to 6.8 mg/mL. Our mAb shared similar basic structure and biological properties with Rituxan. Most importantly, our mAb exhibited greater ADCC effector function than that of Rituxan, which was most likely owing to the afucosylated feature of our mAb, the major difference between our mAb and Rituxan. Thus, our study offers a promising approach for a new generation of anti-CD20 mAb with higher productivity and lower manufacturing cost than Rituxan.

To obtain highly pure mAb for future use, a feasible procedure has been developed, which consisted of two chromatography steps. Success of protein A affinity chromatography relied on the specific binding between the mAb Fc region and Protein A at a low pH, resulting in >93% purity^25^. The anion-exchange chromatography was designed to further remove residual impurities. Through our procedure IgG can be purified to a final purity of 99.8% with a recovery rate of 75%. Assuming that the average concentration of mAbs in milk is 2.5 g/L and a cow can generally yield 8,000 L of milk per year, each cow could produce more than 15 kg of mAb based on 74% recovery after the purification. Therefore, our transgenic cattle mAb production system shows not only great promise for anti-CD20 mAb manufacturing, but also a potent method for various mAb productions in general with low cost.

MAbs are known for their post-translationally glycosylation, which plays an important role in regulating the mAb functions. Various protein expression systems are known to have different glycosylation patterns, affecting the safety and efficacy of the recombinant mAbs, including bioactivity, immunogenicity and pharmacokinetics. Bovine milk contains different types of oligosaccharides, with a relatively high abundance of sialylated and mannosed oligosaccharides and low amount of fucosylated glycans^26^. Due to the fact that glycosylation specificity of bovine mammary gland produced recombinant proteins has not yet been well documented compared to other host cell lines, we characterized the N-glycans of our recombinant mAb and compared them with those of Rituxan. Our results indeed showed distinct N-glycosylation profiles between the CHO- and bovine-derived mAbs. Specifically, the Rituxan glycans are mainly G0F and G1F glycoforms, representing >90% of the fucosylated forms of IgG Fc, which was consistent with a previous report^27^. However, the glycans of our mAb are mainly the G2 glycoform with less fucose and more galactose than the G0F and G1F glycoforms. It was noted that a previous analysis on bovine milk-protein showed the presence of fucosyltransferase, and accordingly, a small quantity of N-linked fucosylated oligosaccharides could be detected. However, we also noted that afucosylated oligosaccharides were not detected in bovine milk in several other studies^28–30^. This discrepancy could be due to the degradation of large fucosylated oligosaccharides by fucosidases, leaving only a small number of fucosylated residues in bovine milk^31^. As a result, low core fucosylation was observed on the recombinant mAb. The afucosylated glycans represented >80% of glycosylation to our recombinant mAb, whereas this value was <5% in Rituxan. Similar scenario was observed in recombinant vs. native human lactoferrin, the lactoferrin expressed in bovine milk had relatively fewer fucosylated residues compared to the native human lactoferrin^32^. These data indicated that the specific glycosyltransferase systems govern the specific mechanisms of protein glycosylation in bovine mammary epithelial cells.

The functional activities are important properties of a potential therapeutic mAb, which must be assessed well before implementation of the mAb. The Fc receptor-mediated effector cell functions, such as ADCC, were believed to be an essential therapeutic mechanism of Rituxan^33,34^. ADCC activity relies on the presence of N-glycans types. Several studies have pointed to the absence of fucose as critical factor for the mAbs biological activities. For example, IgG1 has the improved affinity for FcγRIIIα, confirming the lack of fucose as the reason for high ADCC activity^35–39^. In addition, a new glyco-engineered anti-CD20 mAb drug that was approved by the US FDA has the superiority partly due to the absence of fucosylated sugars^40^. We wish to highlight that our anti-CD20 mAb exhibited a significantly stronger binding affinity for FcγRIIIα and greater ADCC activity than Rituxan. These findings are supported by a previous study that an afucosylated anti-CD20 mAb showed an increased affinity for FcγRIIIα and increased ADCC activity. Besides, other glycans also have the impact on ADCC activity, such as high mannose glycans increasing ADCC activity but sialylation reducing ADCC activity^41,42^. In our study, although the mannosed and sialylated glycans were both higher than those of Rituxan, the recombinant mAb still has greater ADCC activity than Rituxan, which may be due to the fact that the afucosylated glycan in recombinant mAb accounted for the principal components of glycosylation.

Apart from the effector functions of ADCC, the serum half-life and immunogenicity of therapeutic antibodies were also considered a glycosylation-related quality control on safety and potency. We mentioned that, in our study, we found considerable terminal mannose residues such as Man5, Man6 and Man8, which may associated with the clearance rates of therapeutic antibody. Some of the evidences demonstrated that therapeutic mAbs containing high-mannose residues were cleared more rapidly than other glycans^43^. However, other studies have indicated no significant difference in the clearance rates for high-mannose glycans^41^. Besides, another study showed antibody clearance was not significant affected by the glycan structure but glycan cleavage^44^. Above all, the definitive conclusion of the effect of high-mannose glycan on clearance mechanism need to be further confirmed. Therefore, determination of high-mannose content should be considered as a key therapeutic antibody quality control in views of its potential effects on the pharmacokinetic properties. As for the sialylated oligosaccharides, there are two kinds of sialic acid, including the mouse-type N-glycolyneuraminic acid (NGNA) and the human-type N-acetylneuraminic acid (NANA). Previously study has demonstrated that NGNA has an immunogenic risk to patients, while NANA has low hypersensitivity reactions^45^. Our recombinant mAb contained only NANA glycan without NGNA, which is the normal human-type sialylation with low potential immunogenicity. Anyhow, the immunogenicity study must be demonstrated to ensure the safety of recombinant mAbs.

In consistence with above data, a single administration of our recombinant anti-CD20 mAb in SCID/Raji mice (100 μg/mouse) led to complete tumour regression, indicating high efficacy of our mAb *in vivo*. This result further confirmed notion that the unique glycosylation pattern in our mAb from the transgenic cattle resulted in a higher efficacy than Rituxan. Considering the fact that two recombinant proteins derived from the milk of transgenic rabbits and goats, respectively, have been commercialized, the general guidance for the production and downstream processing of recombinant proteins are already available through publications issued by the US DA, the US FDA, and the EMA. Thus, the prospects of producing our recombinant proteins in the mammary glands of transgenic cattle are promising.

In summary, we have generated transgenic cattle expressing high levels of anti-CD20 mAb in their milk. The bioactivities of the anti-CD20 mAb, including its antigen-binding activity and CDC, were comparable to those of Rituxan. Noteworthy, the milk-derived mAb showed stronger ADCC activity than Rituxan, most likely attributed to the unique afucosylated glycanson our milk-derived mAb. Taken together, this expression system offers an effective approach to meet the ever-growing market demand for larger quantities of therapeutic antibodies.

## Electronic supplementary material

Supplementary Information

## Data Availability

The authors declare that all data of this study are available from the corresponding author upon reasonable request.
