# Supplementary material for: A novel glycosylated anti-CD20 monoclonal antibody from transgenic cattle
Source: Sci Rep. 2018 Sep 4;8:13208. doi: 10.1038/s41598-018-31417-2 (PMC6123398; doi:10.1038/s41598-018-31417-2)

A novel glycosylated anti-CD20 monoclonal antibody from transgenic cattle

Ran Zhang[1], Chenjun Tang[1], Huaizu Guo[2], Bo Tang[3], Sheng Hou[2], Lei Zhao[4], Jianwu Wang[3], Fangrong Ding[1], Jianmin Zhao[3], Haiping Wang[1], Zhongzhou Chen[1], Yunping Dai[1*], Ning Li[1*]

[1]State Key Laboratory for Agrobiotechnology, College of Biological Sciences, China Agricultural University, Beijing 100194, China

[2]State Key Laboratory of Antibody Medicine and Targeted Therapy, Shanghai Key Laboratory of Cell Engineering; Shanghai 200433, China

[3]Wuxi KGBIO biotechnology Limited Liability Company, Wuxi 214145, China

[4] National Clinical Research Center for Normal Aging and Geriatric, Institute of Geriatric, PLA General Hospital, Beijing 100853, China

Correspondence and requests for materials should be addressed to Y.D. (email: daiyunping@sina.com) or N.L. (email: ninglcau@126.com)

Figure S1 Verification of transgene integration. (a) Image of transgenic cattle expressing the recombinant anti-CD20 mAb at the farm. (b) PCR detection of the integration sites. The product for the wild-type sequence was 671 bp, whereas the product for the 3′ flanking region of the transgenic sequence was 405 bp. (c, d) Detection of the transgene location in the transgenic cattle by the GTG-binding pattern of metaphase spreads before hybridization (c) and the same metaphase spreads after FISH (d). The arrows indicate the transgene integration site in chromosome 3.

Figure S2 Verification of the transgene genetic stability of transgenic offspring. (a) PCR detection of the transgenes in transgenic offspring. Marker, 1-kb DNA ladder; P-LC, positive plasmid containing the LC-encoding sequences; P-HC, positive plasmid containing the HC-encoding sequences; WT, genomic DNA of wild-type cattle; genomic DNA of transgenic cattle indicated by 1321, 1325 and 0220. The amplified products for the HC- or LC-encoding sequences were 1.5 kb and 0.8 kb, respectively. (b, c) Expression of the recombinant anti-CD20 mAb in the milk of transgenic offspring, as characterized by SDS-PAGE (b) and western blotting under reducing conditions (c). WT, milk from wild-type cattle. PC, purified human IgG.

Figure S3 Peptide mapping of the HC of the recombinant anti-CD20 mAb and Rituxan. Peptide sequences highlighted in red indicate the allotype of the recombinant mAb. The dashes represent amino acids that are identical to those of Rituxan.

Figure S4 SPR analysis of the binding affinity of the recombinant anti-CD20 mAb to human FcγRIIIa. The sensorgrams obtained for the surface blank and buffer blank control were subtracted from the sensorgrams obtained for the antibodies to yield the

curves shown here. (a, b) Interaction between the recombinant anti-CD20 mAb and FcγRIIIa-Phe158 (a) or FcγRIIIa-Val158 (b). The concentrations of the recombinant anti-CD20 mAb are 3.333, 10, 30, 90, 270, and 810 nM, as indicated. (c, d) Interaction between Rituxan and FcγRIIIa-Phe158 (c) or FcγRIIIa-Val158 (d). The concentrations of Rituxan are 30, 90, 270, 810, 2430, 7,290, and 21,870 nM, as indicated.

Table S1. The whole N-glycan profiles of recombinant anti-CD20 mAb.

Milk mAb							
**RT (min)**	**Short name**	**Composition**	**Structura scheme**	**Expected mass (Da)**	**Observed mass (Da)**	**Δmass (ppm)**	**FLR Area%**
15.09	**G0-GlcNAc**	Hex3HexNAc3	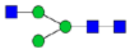	1234.4832	1234.478	-4.21	0.13
18.56	**G0**	Hex3HexNAc4	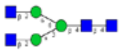	1437.5625	1437.558	-3.13	0.53
18.83	/	Hex5HexNAc2	/	1355.5095	1355.234	-203.29	0.19
20.52	**G1-GlcNAc**	Hex4HexNAc4	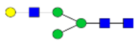	1396.5360	1396.537	0.72	0.94
21.00	**G0F**	Hex3HexNAc4 DHex1	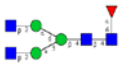	1583.6205	1583.618	-1.58	0.57
22.27	**Man5**	Hex5HexNAc2	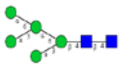	1355.5095	1355.496	-9.96	7.89
23.09		Hex4HexNAc4		1599.6154	1599.608	-4.63	2.71
23.39	**G1**	Hex4HexNAc4	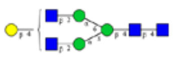	1599.6154	1599.603	-7.75	0.54
23.70		Hex4HexNAc4		1599.6154	1599.609	3.98	0.68
25.17	**Man5Gn**	Hex5HexNAc3	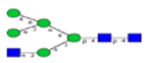	1558.5888	1558.581	5.02	3.96
26.01	**G1F**	Hex4HexNAc4 DHex1	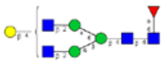	1745.6733	1745.664	-5.33	1.28
26.28	/	Hex4HexNAc5	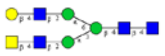	1802.6947	1802.703	-4.58	0.38

Milk mAb							
**RT (min)**	**Short name**	**Composition**	**Structura scheme**	**Expected mass (Da)**	**Observed mass (Da)**	**Δmass (ppm)**	**FLR Area%**
26.91	**Man6**	Hex6HexNAc2	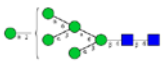	1517.5623	1517.573	7.05	6.72
27.29		Hex6HexNAc2		1517.5623	1517.547	-10.08	2.33
27.97	**G2**	Hex5HexNAc4	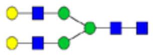	1761.6682	1761.662	-3.52	36.04
29.07	/	Hex6HexNAc3	/	1720.6416	1720.651	-5.44	1.66
29.99	**G2F**	Hex5HexNAc4 DHex1	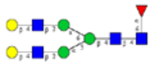	1907.7261	1907.703	-12.11	12.95
30.87	/	Hex5HexNAc3 NANA	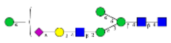	1849.6842	1849.767	-44.74	1.69
31.31	**Man7**	Hex7HexNAc2	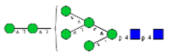	1679.6151	1679.689	44.00	3.13
31.63		Hex7HexNAc2		1679.6151	1679.685	41.61	1.02
32.02		Hex7HexNAc2		1679.6151	1679.672	33.88	1.30
32.92	**G2+NeuAc1**	Hex5HexNAc4 NeuAc1	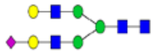	2052.7636	2052.782	8.96	0.90
33.35	/	Hex7HexNAc3	/	1882.6954	1882.689	2.91	1.41
34.13	/	Hex6HexNAc3NeuAc1	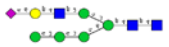	2011.7371	2011.719	-9.00	1.71
34.75			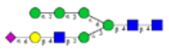	2011.7371	2011.724	6.49	1.95

Milk mAb							
**RT (min)**	**Short name**	**Composition**	**Structura scheme**	**Expected mass (Da)**	**Observed mass (Da)**	**Δmass (ppm)**	**FLR Area%**
35.59	**Man8**	Hex8HexNAc2	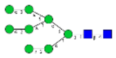	1841.6679	1841.660	-4.29	4.82
36.09	/	Hex6HexNAc4NeuAc1	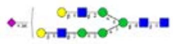	2214.8164	2214.794	-10.11	0.29
36.56				2214.8164	2214.805	-5.15	0.27

Table S2. The whole N-glycan profiles of Rituxan.

Rituxan							
**RT (min)**	**Short name**	**Composition**	**Structura scheme**	**Expected mass (Da)**	**Observed mass (Da)**	**Δmass (ppm)**	**FLR Area%**
18.33	**G0F-GlcNAc**	Hex3HexNAc3 DHex1	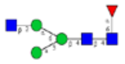	1380.5411	1380.548	5.00	0.79
19.07	**G0**	Hex3HexNAc4	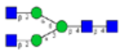	1437.5625	1437.549	-9.39	1.00
21.50	**G0F**	Hex3HexNAc4 DHex1	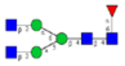	1583.6205	1583.636	9.79	43.58
21.75		Hex3HexNAc4 DHex1		1583.6205	1583.632	7.26	0.50
22.74	**Man5**	Hex5HexNAc2	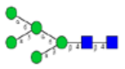	1355.5095	1355.512	1.84	1.27
23.61	**G1F-GlcNAc**	Hex4HexNAc3 DHex1	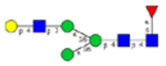	1542.5939	1542.597	2.01	1.03
25.74	**(1,6)G1F**	Hex4HexNAc4 DHex1	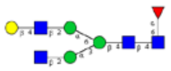	1745.6733	1745.593	-46.00	30.54
26.40	**(1,3)G1F**	Hex4HexNAc4 DHex1	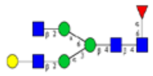	1745.6733	1745.628	-25.95	10.79
28.85	**G1F-GlcNAc +NeuAc1**	Hex4HexNAc3 DHex1NANA1	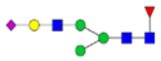	1833.6893	1833.834	78.91	0.20
30.34	**G2F**	Hex5HexNAc4 DHex1	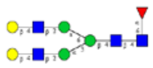	1907.7261	1907.715	-5.82	7.71

Rituxan							
**RT （min）**	**Short name**	**Composition**	**Structura scheme**	**Expected mass (Da)**	**Observed mass (Da)**	**Δmass （ppm）**	**FLR Area%**
32.86	**G2F+ NeuAc1**	Hex5HexNAc4 DHex1NeuAc1	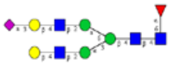	2198.8215	2198.805	-7.50	1.01
35.70	**G2F+ NeuAc2**	Hex5HexNAc4 DHex1NeuAc2	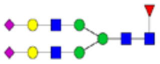	2489.9169	2489.917	0.04	0.41

Table S3. Data collection and refinement statistics for the anti-CD20 Fc fragment*

	Anti-CD20 Fc fragment (PDB: 5IW3)	Anti-CD20 Fc fragment (PDB: 5IW6)
**Data collection**		
Wavelength	0.97915	0.97915
Space group	$C222_1$	$P2_12_12_1$
Cell dimensions		
a, b, c (Å)	64.16, 143.10, 56.56	49.45, 80.00, 139.32
α, β, γ (˚)	90.0, 90.0, 90.0	90.00, 90.00, 90.00
Resolution (Å)†	50.0–2.05 (2.09–2.05)	50.0-2.34 (2.38-2.34)
R_{sym} (%)	7.6 (37.2)	8.7 (57.1)
I/σ	14.0 (2.9)	32.1 (2.1)
Completeness (%)	98.1 (82.6)	99.9 (100.0)
Total No. of reflections	132524	677459
Unique reflections	17005	24182
Redundancy	3.6 (3.6)	6.8 (6.3)
**Refinement**		
Resolution (Å)	50.0-2.05 (2.10–2.05)	50.0-2.34 (2.40-2.34)
No. of reflections	15767 (1095)	22794 (1606)
$R_{\mathrm{work}}/R_{\mathrm{free}}$ (%)	18.9/20.7 (24.1/24.3)	22.4/25.9 (31.9/32.9)
No. of atoms		
Protein	1720	3379
Ligand/ion	23	0
Water	245	107
B-factors (Å^2)		
Protein	26.89	60.75
Ligand/ion	37.30	0
Water	40.40	51.22
RMSDs		
Bond lengths (Å)	0.009	0.010
Bond angles (º)	1.44	1.47
Ramachandran Plot (%)‡	95.0/5.0/0/0	91.8/7.7/0.6/0

*Three crystallization experiments were performed for each structure. †Statistics for the highest resolution shell.

$R_{sym}=\sum_h\sum_i|I_{h,i}-I_h|/\sum_h\sum_i I_{h,i}$, where I_h is the mean intensity of the *ith* observation of the symmetry-related reflections of *h*.

‡Residues in the most favoured, additional allowed, generously allowed, and disallowed regions of the Ramachandran plot.

Table S4. Binding affinity of the recombinant anti-CD20 mAb for human FcγRIIIa.

	158Val/Phe	K_a (1/Ms)	K_d (1/s)	K_D (M)
Rituxan	Val	4.6×10^{4}	4.3×10^{-2}	$9.4\pm0.02\times10^{-7}$
Recombinant mAb	Val	1.7×10^{5}	1.0×10^{-2}	$5.9\pm0.01\times10^{-8}$
Rituxan	Phe	2.5×10^{4}	1.7×10^{-1}	$6.8\pm0.02\times10^{-6}$
Recombinant mAb	Phe	2.1×10^{5}	2.7×10^{-2}	$1.3\pm0.02\times10^{-7}$

Figure S1

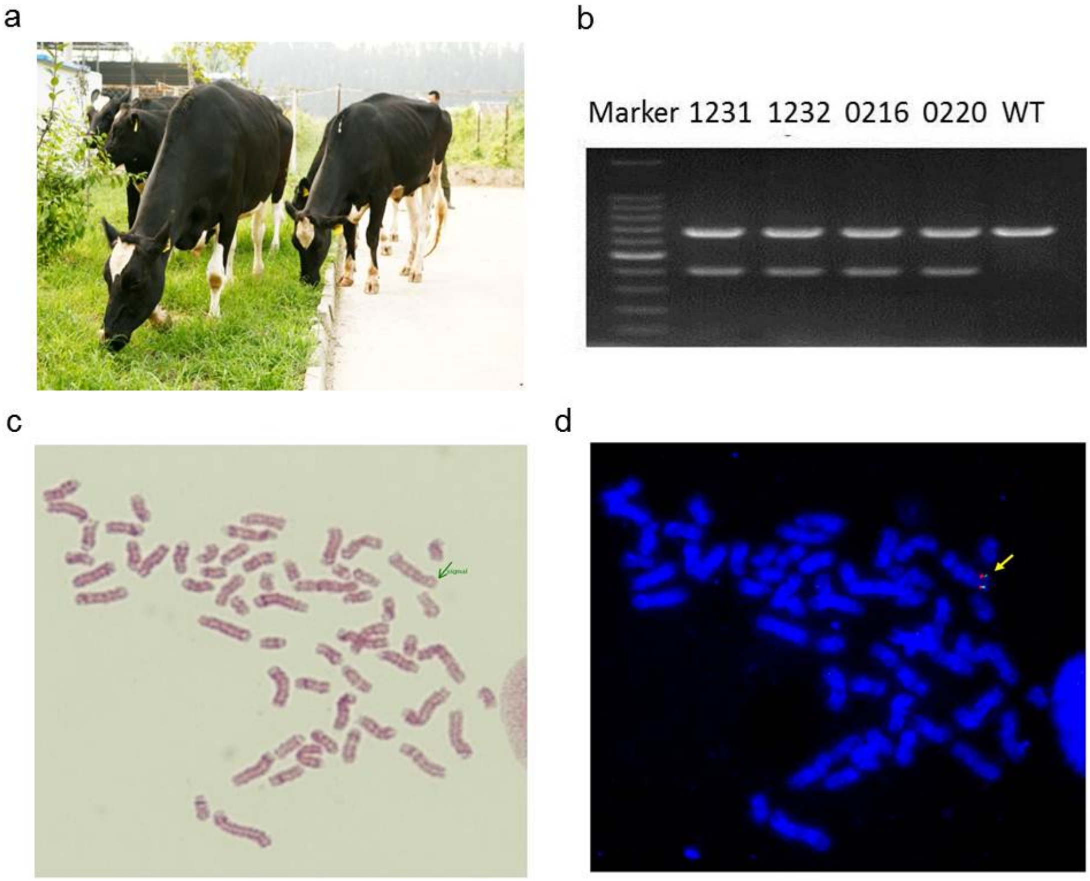

Figure S2

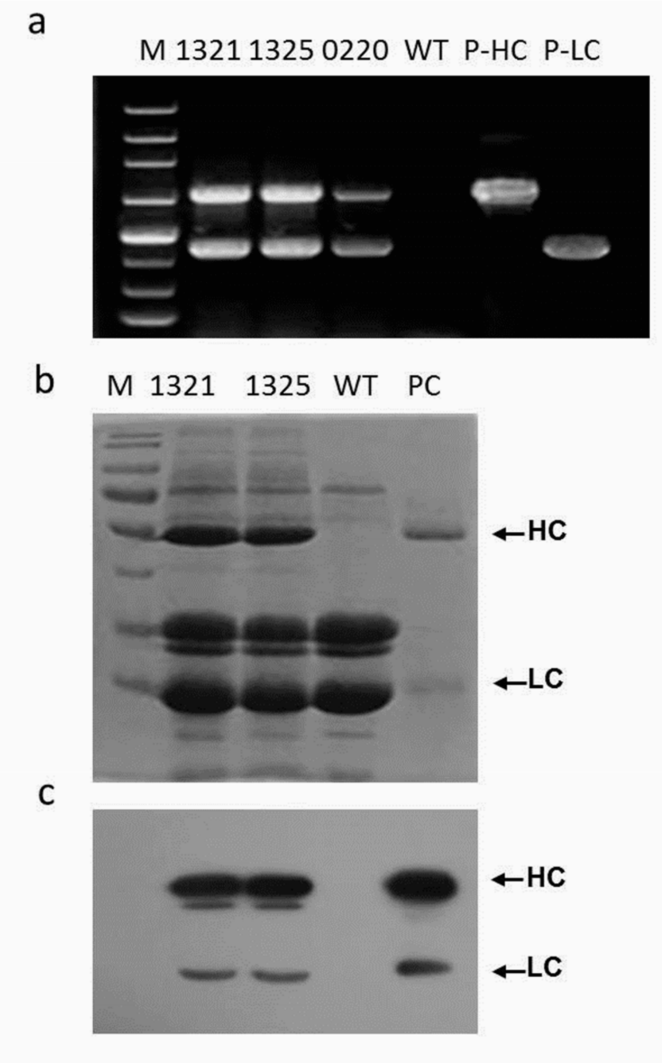

Figure S3

```
N-terminus
  1  QVQLQQPGAE LVKPGASVKM SCKASGYTFT SYNMHWVKQT PGRGLEWIGA
 51  IYPGNGDTSY NQKFKGKATL TADKSSSTAY MQLSSLTSED SAVYYCARST
101  YYGGDWYFNV WGAGTTVTVS AASTKGPSVF PLAPSSKSTS GGTAALGCLV
151  KDYFPEPVTV SWNSGALTSG VHTFPAVLQS SGLYSLSSVV TVPSSSLGTQ
201  TYICNVNHKP SNTKVDKKAE PKSCDKTHTC PPCPAPELLG GPSVFLFPPK
     ---------- -------RVE ---------- ---------- ----------
251  PKDTLMISRT PEVTCVVVDV SHEDPEVKFN WYVDGVEVHN AKTKPREEQY
301  NSTYRVVSVL TVLHQDWLNG KEYKCKVSNK ALPAPIEKTI SKAKGQPREP
351  QVYTLPPSRD ELTKNQVSLT CLVKGFYPSD IAVEWESNGQ PENNYKTTPP
     ---------E EM-------- ---------- ---------- ----------
401  VLDSDGSFFL YSKLTVDKSR WQQGNVFSCS VMHEALHNHY TQKSLSLSPG
451  K                                             C-terminus
```

Figure S4

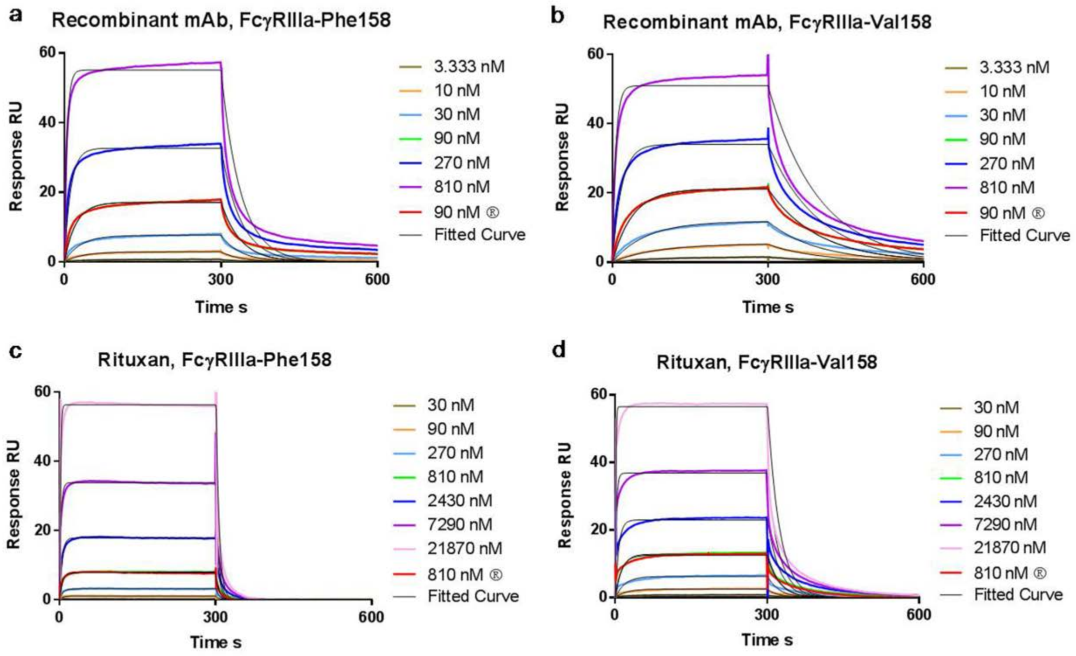

a
Recombinant mAb, FcγRIIIa-Phe158
Response RU
Time s
3.333 nM
10 nM
30 nM
90 nM
270 nM
810 nM
90 nM ®
Fitted Curve
b
Recombinant mAb, FcγRIIIa-Val158
Response RU
Time s
3.333 nM
10 nM
30 nM
90 nM
270 nM
810 nM
90 nM ®
Fitted Curve
c
Rituxan, FcγRIIIa-Phe158
Response RU
Time s
30 nM
90 nM
270 nM
810 nM
2430 nM
7290 nM
21870 nM
810 nM ®
Fitted Curve
d
Rituxan, FcγRIIIa-Val158
Response RU
Time s
30 nM
90 nM
270 nM
810 nM
2430 nM
7290 nM
21870 nM
810 nM ®
Fitted Curve

Full-length gels and blots for SDS-PAGE and Western blotting experiments.

Figure 1(d)

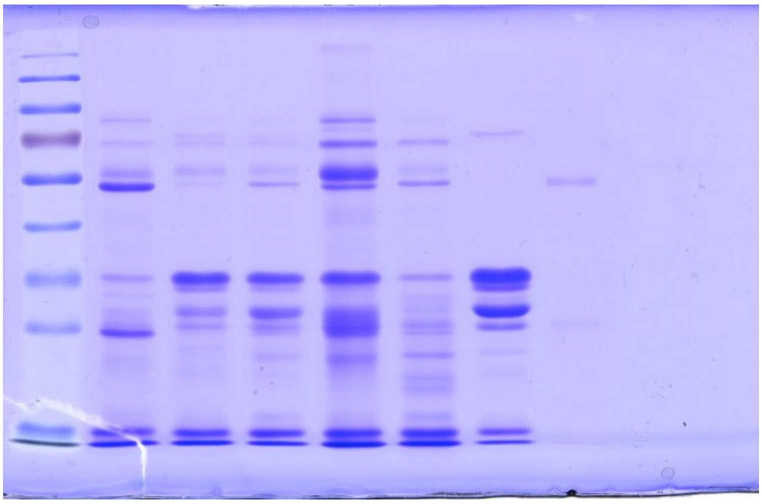

Figure 1(e)

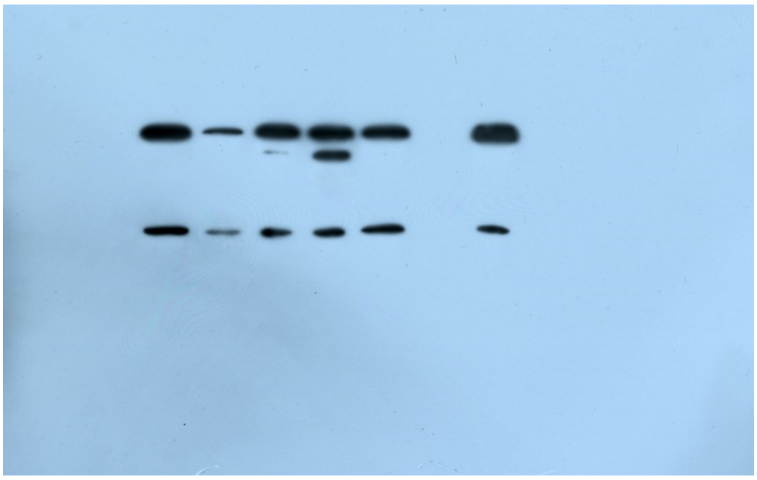

Figure 1(f)

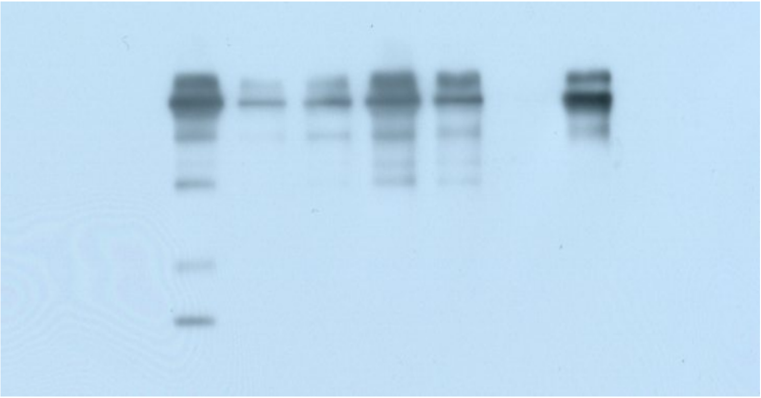

Figure 2(c)

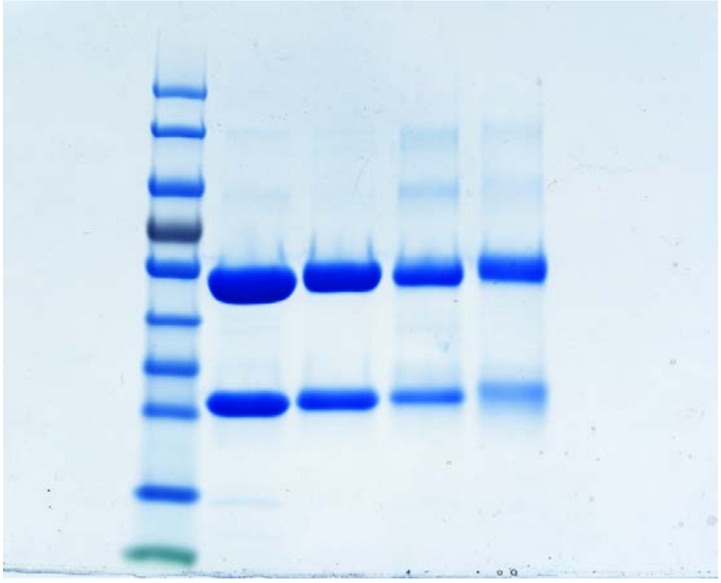

Figure 2(d)

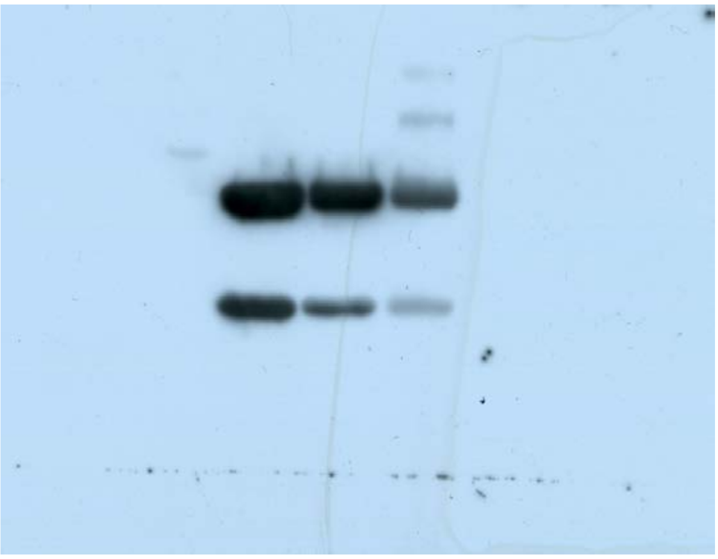

Supplement: Supplementary file 1 — Supplementary Information [file 41598_2018_31417_MOESM1_ESM.pdf]
